# Neighborhood Environment Is Associated with Overweight and Obesity, Particularly in Older Residents: Results from Cross-Sectional Study in Dutch Municipality

**DOI:** 10.1007/s11524-015-9991-y

**Published:** 2015-10-09

**Authors:** Polina Putrik, Ludovic van Amelsvoort, Nanne K. De Vries, Suhreta Mujakovic, Anton E. Kunst, Hans van Oers, Maria Jansen, IJmert Kant

**Affiliations:** Department of Health Promotion, School for Public Health and Primary Care (CAPHRI), Maastricht University, Maastricht, The Netherlands; Academic Collaborative Centre for Public Health Limburg, Public Health Service Southern Limburg, Geleen, Netherlands; Department of Epidemiology, School for Public Health and Primary Care (CAPHRI) Maastricht University, Maastricht, The Netherlands; Department of Public Health, Academic Medical Center, Amsterdam, The Netherlands; National Institute of Public Health and the Environment (RIVM), Bilthoven, The Netherlands; Tranzo Scientific Centre for Care and Welfare, Tilburg University, Tilburg, The Netherlands; Department of Health Services Research, School for Public Health and Primary Care (CAPHRI), Maastricht University, Maastricht, The Netherlands

**Keywords:** Neighborhood, Social and physical environment, Obesity, Overweight, Socioeconomic inequalities

## Abstract

We explored whether overweight and obesity were associated with the physical and social environment at neighborhood level. Data from Maastricht municipality survey (the Netherlands) were used (*n* = 9771 adults). Multinomial regression models were computed (outcome being normal weight, overweight, or obese). We found inconsistent associations between neighborhood social and physical environment characteristics and overweight and obesity in the total sample. The effects were more consistent and stronger for older residents (>65) and obesity as an outcome. Better scores on traffic nuisance, green space, social cohesion, nuisance, and safety were associated with lower odds of obesity among elderly (OR ranged between 0.71 [95 % CI 0.44 to 0.93] to 0.85 [95 % CI 0.74 to 0.96] for each point of improvement in neighborhood social and physical environment (scale 0–10)). We showed that there are neighborhood-level factors that are associated with obesity, particularly in elderly residents. These could be targeted in preventive strategies outside health care settings.

**Electronic supplementary material:**

The online version of this article (doi:10.1007/s11524-015-9991-y) contains supplementary material, which is available to authorized users.

## Introduction

The worldwide prevalence of obesity has nearly doubled since 1980 according to the World Health Organization.^1^ In the Netherlands, the prevalence of overweight and obesity increased from 33 % in 1981 to 48 % in 2012 and the prevalence of obese persons rose from 5 to 12 %.^2^ It is well established that an increase in obesity rates leads to a rapid growth of health care costs associated with diabetes, cardiovascular diseases, musculoskeletal conditions, and some forms of cancer.^1^ It has been suggested that four main factors relate to obesity: built environment, social environment, individual behavior, and individual genetic factors.^3^ In this context, experts talk about obesogenic environments, i.e., environments that promote individual obesity-enhancing behaviors and thus obesity.^3^^,^^4^ For example, the immediate environment can increase or reduce opportunities for healthy eating as well as stimulate or discourage physical activity (PA). The effect of the abovementioned factors are suggested to be especially pronounced in those persons who are genetically predisposed to obesity.^3^

While many interventions have been developed to address obesity at an individual level (e.g., interventions targeting individual behavior related to nutrition and physical activity, but also antiobesity drugs or surgery^5^^–^8), these are not able to control the emerging obesity pandemic.^9^^,^^10^ In a view of this growing consensus, it has been suggested that approaches that target community (as opposed to individual) risk factors could add to traditional individual-based obesity interventions.^11^ Neighborhood socioeconomic deprivation has previously been shown to be associated with obesity in developed countries, including the Netherlands.^5^^,^^12^^–^16 A more specific focus on neighborhood determinants of obesity may offer more “upstream” (population level) preventive strategies and reach larger population groups.^11^ In view of this, there is an increasing interest in the potential of population-level interventions to prevent overweight and obesity. Such interventions would require intervening in the social and physical environment in which individuals live.

To our knowledge, only a few studies have explored a broad range of neighborhood characteristics and their relative importance for overweight and/or obesity, while other studies have focused on one or a few particular aspects of neighborhood in relation to excessive weight. The majority of such evidence has come from the USA, and study findings have been inconclusive. While some studies found that the built environment, green space, neighborhood density, walkability, distance to parks, traffic, and cleanliness of the neighborhood tended to be associated with overweight and/or obesity^4^^,^^5^^,^^11^^–^17, others found no proof for many of these associations.^18^^,^^19^ Among studies performed in the Netherlands, only one focused on specific aspects of the neighborhood environment in relation to obesity as an outcome; it found that less crime was positively associated with use of active transportation and more cycling, in turn, was associated with lower BMI among elderly male residents.^20^ The relationship between neighborhood characteristics and physical activity as an outcome has been more frequently explored, but findings remain inconsistent.^21^^–^26 So far, therefore, the available evidence about links between the neighborhood environment and obesity appears to be largely indirect and inconclusive. Gender has been pointed out as a potential effect moderator by several studies, but evidence regarding the moderating effect of other demographic or socioeconomic factors is limited, and further examination of potential moderators is warranted.^11^^,^^25^^,^^27^ While problems of potential selection mechanisms (in addition to or instead of causal relationships) in the interpretation of associations between neighborhood environment and health are difficult to overcome,^28^ investigating these relationships for different age groups could be particularly informative, as selection may be more likely to occur among younger persons who are more mobile. While the body of the international literature on the relationship between neighborhood and obesity has expanded in the last decade, contradictory findings and the dependence of findings on the particular context invite new studies to strengthen the available evidence.

Municipalities in the southern part of the Dutch province of Limburg have developed a long-term strategy that recognizes that both individual factors and the residential environment (regarding domains such as safety, spatial planning, care, and education) affect the health of residents.^29^^,^^30^ Municipal authorities in Southern Limburg (but also in the Netherlands as a whole) have a tradition of monitoring physical and social environments to tailor their policies, and therefore have large amounts of routinely collected data available. This makes this region a suitable area to identify neighborhood effects on overweight and obesity.

The objective of this study was to explore whether overweight and obesity are associated with the specific characteristics of the physical and social neighborhood environment, after accounting for individual demographic factors and socioeconomic status, and whether the association depends on age, gender, and socioeconomic categories. We hypothesize that better scores on the neighborhood environment measures are associated with lower rates of excessive weight, acting via increased physical activity as well as reducing the stress levels also shown to be associated with obesity.^13^^,^^31^^,^^32^ Further, we expect that these effects are more visible in female and older residents who generally spend more time in the neighborhood, and in lower SES residents who may have fewer resources to compensate for an unfavorable living environment. Results of our study could inform the policy-makers as to which of the modifiable neighborhood risk factors could be considered in order to, in the long run, reduce the number of obese and overweight persons.

## Methods

### Source of Data

We used cross-sectional survey data from the local authorities of Maastricht, the largest municipality in Southern Limburg (122,488 inhabitants, 60.03 km^2^^33^). This survey is conducted biannually by the municipal authorities among noninstitutionalized inhabitants and uses a probability sample. A questionnaire is sent to a household, and the person whose birthday comes first after the date on which the questionnaire was received is asked to complete it. The survey includes questions on aspects of the neighborhood environment, such as the quality and accessibility of facilities, safety and nuisance, perceptions of traffic and the built environment, aspects of social capital, health status, demographic and socioeconomic background, and weight and height. The survey is conducted among adults aged 18 years or over. We used the data from the 2010 survey.

Thirty-nine neighborhoods (as defined by “buurt code” by Statistics Netherlands^34^) were included in the analyses (150 to 6305 inhabitants per neighborhood). Very small neighborhoods with less than 100 residents (3 neighborhoods) were excluded in advance from the sample.

### Variables

Data were collected on demographics (age and gender), socioeconomic status (education and income), physical activity, height and weight, mental health, and smoking status. Socioeconomic status was measured by level of educational achievement and income group. The six education categories mentioned in the questionnaire were classified as low educational level (primary education, lower vocational education, pre-vocational secondary education), secondary education (secondary vocational education, senior general secondary education/pre-university education), or high educational level (Bachelor’s degree and higher). Income group was defined as low, middle, or high income and was self-reported by the respondents.

### Outcome Variable

Body mass index (BMI) was a variable of interest. BMI was computed from self-reported weight (kg) and height (cm). BMI was classified into normal weight (18.5 ≥ BMI < 25), overweight (25 < BMI ≤ 30), and obese (BMI > 30).^19^^,^^35^ Persons who were underweight (BMI < 18.5) were excluded from the statistical analyses because of the low numbers (*n* = 108 (1.1 %)) as well as the fact that different mechanisms can be assumed to underlie underweight and overweight/obesity.

### Statistical Analyses

First, we created 12 aggregated measures of neighborhood environment from 52 original survey questions. Factor analysis was used to define aggregated neighborhood characteristics of physical or social environment, which were then used in the current analyses. Scale reliability analyses were performed for identified factors to determine the internal consistency between the indicators grouped in each factor (Cronbach’s alpha >0.7). Next, each factor was labeled based on face validity upon a consensus among the project group members. A total score was computed for each factor. To adjust for different numbers of answering alternatives, each individual component was recoded to a scale of 0 to 10, where 10 corresponded to the most favorable answer (e.g., for the question with 5 answer categories, the following scores would be assigned: “absolutely not satisfied” = 0, “not satisfied” = 2.5, “not dissatisfied/not satisfied” = 5, “satisfied” = 7.5, “very satisfied” = 10). The total score for a factor was computed as the mean of the individual components, which also took values from 0 to 10. If one individual item score was missing, the mean of the remaining components was taken. If more than one individual component was missing, the total score of the factor was considered to be missing. In total, 12 factors were defined, namely quality and availability of parking facilities, quality and availability of daily shopping facilities, reachability of facilities for daily use, traffic nuisance, quality and availability of green space, social cohesion, general nuisance by people, general feeling of safety, thefts, neighborhood aesthetics (cleanliness), damage to physical environment, and nuisance by drunk people (Table 2).^36^

Next, we fitted multinomial logistic regression models to explore the relationship between each computed measure of physical and social environment and the odds of having normal weight, being overweight, or being obese. Each mean aggregated indicator of a neighborhood environment characteristic was modeled separately as an independent variable, in view of the high correlation between the aggregated indicators and the limited power of the model (total number of neighborhoods *n* = 39). Each regression model was adjusted for age, gender, and education. As a sensitivity analysis, models were repeated adjusting additionally for income. To explore whether the associations of neighborhood environment characteristics with overweight and obesity depended on demographic or socioeconomic characteristics of the individuals, interactions between individual- and neighborhood-level characteristics (in a fully adjusted model) were tested (cutoff *p* value for interaction term 0.05). Analyses were performed on the complete cases available for each model. Statistical significance was assumed at the 0.05 level. Statistical package STATA 12 was used.^37^

## Results

### Study Population

In total, 9771 residents of Maastricht were included in the study (response rate 25 %). The mean age of the respondents was 55 years, and 50 % were male. Thirty-nine percent of respondents were highly educated while 33 % had the lowest level of educational achievement. Most of the respondents (51 %) classified themselves in the middle income group. In total, half of the respondents met the national norm for physical activity (54 %), and the average respondent reported engaging in at least moderate physical activity for a minimum of 30 min a day on 4.1 days per week. Almost half (44 %) had a normal weight, and 39 and 13 % were overweight and obese, respectively (Table 1, Online appendix Table 1).

**TABLE 1:** Sociodemographic and health-related lifestyle characteristics of the sample

	At individual level	At neighborhood level
Variable	Mean (SD) [min–max], *N* (%)	Min–max of the neighborhood level indicators^a^
Age	55.4 (15.8) [18–98]	46.3–65.0
*Missing n*	*179* (*1.8*)	
Gender		
Male	4862 (49.8)	41.5–58.1 %
*Missing n*	*171* (*1.8*)	
Education		
Low	3255 (33.3)	9.0–61.5 %
Secondary	2277 (23.3)	4.2–36.8 %
High	3845 (39.4)	13.2–71.0 %
*Missing n*	*394* (*4.0*)	
Income (self-classified)		
Low income	1952 (20.0)	1.0–43.2 %
Middle income	4958 (50.7)	24.4–71.3 %
High income	2118 (21.7)	4.9–70.4 %
*Missing n*	*743* (*7.6*)	
Obesity		
Normal weight (18.5 ≤ BMI < 25)	4290 (43.9)	33.0–59.5 %
Overweight (25≤ BMI < 30)	3783 (38.7)	32.9–49.5 %
Obese (BMI > 30)	1299 (13.3)	3.0–25.3 %
Missing n	*399* (*4.1*)	

^a^Range of means among 39 neighborhoods for continuous variables, and range of (lowest and highest) percentage per neighborhood for categorical variables

At neighborhood level, substantial differences in socioeconomic characteristics were observed. The percentage of low educated individuals ranged from 9 to 62 %, and the percentage of residents who perceived their income as low ranged from 1 to 43 %. Substantial differences were found in overweight and obesity rates (33–50 and 3–25 %, respectively). Mean scores on characteristics of neighborhood environment are presented in Table 2.

**TABLE 2:** Aggregated indicators of social and physical environment

Neighborhood environment indicator	Neighborhood scores, mean (SD) [min–max]
Physical environment	
Quality and availability of parking facilities	5.42 (0.71) [3.13–6.94]
Quality and availability of daily shopping facilities	7.03 (1.44) [0.00–8.59]
Reachability of facilities for daily use	6.49 (1.04) [1.50–7.78]
Traffic nuisance	5.88 (0.87) [1.00–7.42]
Quality and availability of green space	5.93 (0.47) [4.67–7.50]
Neighborhood aesthetic (cleanliness)	3.89 (0.23) [1.67–5.00]
Damage to physical environment	5.33 (1.01) [2.50–8.19]
Social environment	
Social cohesion	6.93 (0.59) [5.66–8.02]
General nuisance by people	7.67 (1.01) [1.25–9.27]
General feeling of safety	7.58 (0.79) [3.09–8.95]
Thefts	6.13 (0.95) [4.00–8.52]
Nuisance by drunk people	8.42 (1.12) [3.79–10.00]

All aggregated indicators of the neighborhood environment were scored 0 to 10; the higher the score, the more favorable the perception of the situation corresponding to the indicator

### Relationship Between Neighborhood Environment Characteristics and Obesity and Physical Activity

More favorable neighborhood scores regarding quality and availability of daily shopping facilities, reachability of facilities for daily use (such as post office, general practitioner’s office and pharmacy, bank and cash dispenser), and neighborhood aesthetics were significantly associated with lower odds of overweight and obesity, regardless of an individual’s age, gender, and education (Table 3). Respondents living in neighborhoods with better quality and availability of parking facilities and less nuisance by drunk people were found to be at increased risk of obesity (OR 1.13 and OR 1.08, respectively) and overweight (OR 1.10 and 1.09, respectively). Additionally, quality and availability of green space, social cohesion, general nuisance by people, and general feeling of safety were only significantly associated with obesity as an outcome. In general, stronger associations were found for obesity than for overweight, and for two factors (traffic nuisance and thefts), the associations went in opposite directions for obesity and overweight (Table 3). The range of mean scores for different aspects of the environment varied greatly across neighborhoods, but was never less than two points between the most and least disadvantaged neighborhoods, and reached a difference of as much as 8 points on a 0–10 scale (Table 2). An OR of 0.90 for each point of improvement (on a 0–10 scale) thus yielded a decrease in the risk of excessive weight of 19 % (1–0.90^2^) to 43 % (1–0.90^8^) in the neighborhoods that scored best on a particular environmental characteristic.

**TABLE 3:** Association between the characteristics of the neighborhood and overweight and obesity

Characteristic of neighborhood environment^a,b^	Overweight vs normal weight	Obese vs normal weight
	Odds ratio [95 % CI]^c^	
Physical environment		
Quality and availability of parking facilities	**1.10** [**1.04**;**1.18**]	**1.13** [**1.03**;**1.24**]
Quality and availability of daily shopping facilities	**0.94** [**0.91**;**0.97**]	**0.94** [**0.90**;**0.98**]
Reachability of facilities for daily use	**0.94** [**0.90**;**0.98**]	**0.92** [**0.87**;**0.98**]
Traffic nuisance	**1.08** [**1.02**;**1.14**]	**0.91** [**0.84**;**0.98**]
Quality and availability of green space	1.08 [0.98;1.20]	**0.84** [**0.73**;**0.97**]
Neighborhood aesthetics (cleanliness)	**0.81** [**0.66**;**0.99**]	**0.59** [**0.44**;**0.79**]
Damage to physical environment	1.01[0.96;1.05]	0.94 [0.88;1.01]
Social environment		
Social cohesion	1.00 [0.92;1.09]	**0.78** [**0.70**;**0.88**]
General nuisance by people	1.05 [1.00;1.10]	**0.92** [**0.86**;**0.98**]
General feeling of safety	1.06 [1.00;1.13]	**0.87** [**0.80**;**0.95**]
Thefts	**1.07** [**1.02**;**1.13**]	0.95 [0.89;1.02]
Nuisance by drunk people	**1.09** [**1.05**;**1.14**]	**1.08** [**1.01**;**1.15**]

^a^ *n* = 9034

^b^All aggregated indicators of neighborhood environment are scored 0 to 10; the higher the score, the more favorable the perception of the situation corresponding to the indicator

^c^Odds ratios per unit increase of the score, derived from multinomial logistic regression adjusted for individual age, gender, and education category. Estimates with *p* value <0.05 are highlighted in bold

Statistically significant interactions with age were found for nearly all neighborhood characteristics. Stratified analyses across the age groups (young adults (18–40 years), middle-aged (41–65 years), and older adults (>65 years) revealed substantially different patterns. In the younger age groups, the associations were in different directions for obesity and overweight, meaning that a more favorable neighborhood environment was both positively and negatively associated with overweight and obesity, depending on the characteristic. At the same time, among the residents of older ages (>65 years), more favorable physical and social environment characteristics were consistently associated with lower odds of being overweight and obese (Table 4). Reachability of facilities for daily use, traffic nuisance, quality and availability of green space, social cohesion, general nuisance by people, general feeling of safety, and neighborhood aesthetics were among the most prominent characteristics of the neighborhood associated with a lower likelihood of overweight and obesity among the older adults (Table 4).

**TABLE 4:** Association between neighborhood characteristics and overweight and obesity, stratified by age

Characteristic of neighborhood environment^a^	Overweight vs normal weight	Obese vs normal weight	Overweight vs normal weight	Obese vs normal weight	Overweight vs normal weight	Obese vs normal weight
	Odds ratio [95 % CI]^b^					
Age category	18 to ≤40^c^		40 to ≤65^d^		>65^e^	
Physical environment						
Quality and availability of parking facilities	**1.39** [**1.19**;**1.62**]	**1.66** [**1.27**;**2.15**]	**1.11** [**1.02**;**1.23**]	1.11 [0.98;1.26]	0.93 [0.82;1.04]	0.93 [0.79 ;1.10]
Quality and availability of daily shopping facilities	**0.86** [**0.79**;**0.94**]	0.88 [0.77;1.00]	**0.94** [**0.90**;**0.99**]	**0.94** [**0.89**;**0.99**]	0.98 [0.92;1.04]	0.97 [0.89;1.05]
Traffic nuisance	**1.23** [**1.08**;**1.39**]	1.13 [0.92;1.39]	**1.10** [**1.02**;**1.18**]	**0.87** [**0.79**;**0.96**]	0.92 [0.83;1.02]	**0.83** [**0.71**;**0.96**]
Quality and availability of green space	**1.45** [**1.15**;**1.84**]	1.25 [0.84;1.86]	**1.16** [**1.01**;**1.33**]	**0.80** [**0.66**;**0.96**]	**0.79** [**0.66**;**0.96**]	**0.71** [**0.54**;**0.93**]
Damage to physical environment	1.08 [0.97;1.21]	1.03 [0.86;1.24]	1.00 [0.94;1.06]	0.92 [0.85;1.00]	0.97 [0.88;1.07]	0.89 [0.77;1.02]
Social environment						
Social cohesion	1.12 [0.91;1.37]	0.96 [0.68;1.36]	1.05 [0.94;1.17]	**0.72** [**0.62**;**0.84**]	0.87 [0.74;1.01]	**0.80** [**0.64**;**0.99**]
General nuisance by people	**1.19** [**1.07**;**1.33**]	1.15[0.95;1.38]	1.05 [0.98;1.12]	**0.87** [**0.80**;**0.95**]	0.93 [0.85;1.02]	**0.85** [**0.74**;**0.96**]
General feeling of safety	**1.19** [**1.03**;**1.38**]	1.02 [0.81;1.29]	1.08 [1.00;1.18]	**0.83** [**0.74**;**0.93**]	0.92 [0.82;1.04]	**0.83** [**0.70**;**0.98**]
Thefts	**1.19** [**1.06**;**1.34**]	1.17 [0.97;1.41]	**1.08** [**1.01**;**1.15**]	**0.91** [**0.83**;**0.99**]	0.96 [0.87;1.06]	0.89 [0.78;1.03]
Nuisance by drunk people	**1.26** [**1.14**;**1.38**]	**1.48** [**1.21**;**1.80**]	**1.07** [**1.01**;**1.13**]	1.03 [0.95;1.12]	0.94 [0.84;1.06]	0.98 [0.90;1.06]

Stratified analyses are only performed when interaction term was significant (*p* < 0.05)

^a^All aggregated indicators of neighborhood environment are scored 0 to 10; the higher the score, the more favorable the perception of the situation corresponding to the indicator

^b^Odds ratios per unit increase of the score, derived from multinomial logistic regression adjusted for individual gender and education category. Estimates with *p* value <0.05 are highlighted in bold

^c^ *n* = 1728

^d^ *n* = 4935

^e^ *n* = 2371

In females, quality and availability of green space, social cohesion, general nuisance by people, general safety feeling, and thefts were protective factors for obesity, but this did not hold true for males where relationships were either not significant or in the opposite direction (Table 5). No clear patterns in the direction or strength of associations were found between education categories (Online appendix Table 2). Models additionally adjusted for income did not yield different conclusions (results not shown).

**TABLE 5:** Association between neighborhood characteristics and overweight and obesity, stratified by gender

Characteristic of neighborhood environment^a^	Overweight vs normal weight	Obese vs normal weight	Overweight vs normal weight	Obese vs normal weight
	Odds ratio [95 % CI]^b^			
Gender	Females^c^		Males^d^	
Physical environment				
Quality and availability of green space	1.05 [0.91;1.22]	**0.74** [**0.61**;**0.91**]	1.12 [0.97;1.28]	0.95 [0.77;1.16]
Social environment				
Social cohesion	0.91 [0.80;1.02]	**0.69** [**0.59**;**0.82**]	1.09 [0.98;1.23]	0.89 [0.76;1.05]
General nuisance by people	0.99 [0.93;1.07]	**0.87** [**0.79**;**0.95**]	**1.09** [**1.02**;**1.16**]	0.97 [0.88;1.06]
General safety feeling	0.99 [0.91;1.09]	**0.81** [**0.72**;**0.91**]	**1.12** [**1.03**;**1.22**]	0.94 [0.83;1.05]
Thefts	1.04 [0.96;1.12]	**0.89** [**0.80**;**0.98**]	**1.11** [**1.03**; **1.19**]	1.02 [0.92;1.12]

Stratified analyses are only performed when interaction term was significant (*p* < 0.05)

^a^All aggregated indicators of neighborhood environment are scored 0 to 10, the higher the score, the better is the perception of the situation corresponding to the measured indicator

^b^Odds ratios per unit increase of the score, derived from multinomial logistic regression adjusted for individual age and education group. Estimates with *p* value <0.05 are highlighted in bold

^c^ *n* = 4396

^d^ *n* = 4638

## Discussion

The main purpose of the present study was to explore whether obesity and overweight are associated with characteristics of the physical and social neighborhood environment, taking into account individual demographic factors and socioeconomic status, and whether patterns of associations differ for different subgroups in the population.

In the total sample, better quality and availability of daily shopping facilities, reachability of facilities for daily use (such as post office, general practitioner’s office and pharmacy, bank and cash dispenser), and neighborhood aesthetics were associated with lower prevalence of both overweight and obesity (better environment being associated with lower odds of excessive weight). ORs for each point of improvement in the environment on a 0 to 10 scale varied between 0.59 and 0.94 and were statistically significant. Additionally, green space, social cohesion, nuisance, and feelings of safety were only relevant when obesity (BMI > 30) was the outcome. As regards traffic nuisance and thefts, associations went in opposite directions: a poorer score on an environment aspect was associated with less overweight but with more obesity, and reverse associations for both overweight and obesity were found for parking facilities and nuisance by drunk people.

Although findings in general sample were generally somewhat inconclusive and the direction of some associations was counterintuitive and hard to explain, analyses stratified by age revealed that in elderly subsamples (>65) an unfavorable physical and social environment was consistently associated with higher levels of obesity among residents. Older residents were more likely to be obese in less cohesive and unsafe neighborhoods, neighborhoods with more traffic nuisance, and those with poor aesthetics. Among all the characteristics we explored, quality and availability of green space had the strongest association with both outcomes in the elderly. Plausibly, older people spend more time outdoors in neighborhoods with more green space, and this has positive impact on BMI via increased physical activity and even reducing stress levels. Green space quality, social cohesion, and safety were also among factors shown to be protective for obesity in females, in addition to nuisance by neighbors. In males, these relationships were either not significant, or even in reverse direction in case of safety and nuisance. Recent study by Bell et al. has observed that associations between neighborhood characteristics and obesity indicators were evident for women only.^27^ It is possible that females spend more time in the neighborhood due to either lower participation in employment or their caregiving responsibilities.^27^ A stronger association among older residents might also indicate that duration of exposure to neighborhood characteristics is involved, as elderly, on general, have lived longer in their specific neighborhood.

Stafford and colleagues concluded that in England and Scotland a higher level of neighborhood social disorder and poor access to local high street facilities were factors that might promote higher obesity rates among the residents. These factors were also significant in our analyses (although the wording of the questions in our survey was not identical to theirs).^13^ Social nuisances were also observed to be associated with obesity in an earlier study by Poortinga in England.^14^ Recent studies had contradictory findings in respect of social cohesion, e.g., some authors did not find evidence that social cohesion is related to BMI^15^^,^^18^ while Bjornstrom reported that collective efficacy (trust, cohesion, and the willingness to intervene for the common good among residents) “exerts an independent and beneficial effect on obesity”.^16^ In our sample, social cohesion was associated with obesity (but not overweight) and this relationship was more pronounced among older residents.

In agreement with our findings, features of the physical environment were also previously found to be associated with obesity.^15^^,^^17^ Recent studies from New Zealand and Europe (including eight countries), which explored several neighborhood factors in relation to outcomes similar to ours in a general adult population mentioned green space (objectively measured) as one of the most relevant characteristics, together with general neighborhood deprivation and aesthetic features.^5^^,^^38^ While we have observed similar associations in our Dutch sample, we noticed that green space was not equally relevant across age groups, with results being more pronounced for obesity and for middle- and older-aged residents. Powell-Willey et al. observed that perceived traffic nuisance was a risk factor for obesity, while Boehmer et al. reported no such association with an objective traffic safety measure (in a general sample).^15^^,^^17^ We could only confirm this association for older residents. Certain discrepancies in findings may be due to the choice of objective and subjective measures.^25^ Moreover, available studies are not directly comparable due to differences in the measurements reflecting the neighborhood environment and the set of confounders used for adjustment. Nevertheless, our results to a large extent confirm the previous and add to the existing knowledge by refined analyses based on age, gender, and education.

Our finding that associations between most neighborhood environment characteristics and obesity are more relevant for older age categories could be explained by the fact that older people are likely to be exposed to the particular neighborhood environment longer and more intensively, while the younger population, on average, would have lived in the neighborhood for a shorter period, and have been less exposed to the neighborhood factors. Effects found in younger subsamples are also more likely to be the result of selection mechanisms, meaning that the choice to live in a specific neighborhood might be influenced by changing individual or neighborhood factors (although selection cannot be completely ruled out for older ages either). It is important that future studies in the field thoroughly explore the age patterns of the neighborhood effects on excessive weight.^26^

A clear strength of our study is the large sample, which allowed us to go into great detail in exploring the effects of neighborhood characteristics in subgroups, something which was previously noted in the literature as lacking.^11^ Another strong point of our study is the practical decision to reduce the number of routinely asked survey questions to a reasonable number of meaningful neighborhood characteristics.^36^ This approach allowed us to make the best use of the rich data set of the survey, while keeping a manageable number of operational concepts in our analyses. Further, we have related outcomes to the perception of neighborhood conditions as reported by co-residents, instead of the respondents themselves, thus overcoming the “single-source” reporting bias. Ours is the first study in the Netherlands and one of few in the international literature to have operationalized neighborhood environment in a large spectrum of specific characteristics (as opposed to exploring only one or a few characteristics of the neighborhood) and explored the relationships with the risk of both overweight and obesity among the residents.

Our study also has a few notable limitations. First of all, the low response to the survey (25 %) may have biased our findings. While this limitation cannot be disregarded, low response is not necessarily an indicator of poor survey quality.^39^^,^^40^ A comparison of the demographics of the sample with data from Statistics Netherlands shows that our sample, although it had a somewhat higher proportion of higher educated individuals, was comparable to the general Dutch population in terms of age and gender. Secondly, a cross-sectional design is commonly mentioned as a limitation, as it precludes conclusions on causal effects.^25^ Currently, all available evidence on this topic comes from cross-sectional studies, and even these are relatively scarce. Although the risk of confounding by unmeasured individual (e.g., ethnicity or cultural factors) or neighborhood-level factors (such as population density or neighborhood-level socioeconomic status) cannot be ignored, we did adjust our models for important individual demographic and socioeconomic variables, namely age, gender, education, and income. Thirdly, while we were able to explore a considerable number of neighborhood factors in relation to obesity and overweight, our data did not allow us to assess the independent associations and weigh the relative importance of these factors in a multivariable model. Fourthly, there were some issues related to defining the neighborhood boundaries and the fact that respondents may not refer to the same area in their responses, which have been discussed elsewhere.^13^^,^^36^^,^^41^^–^43 A recent Dutch study by Veldhuizen et al. presented an analysis of relationships between socioeconomic variables and health at different area sizes and suggested that administrative areas that are defined on the basis of socioeconomic and geographic criteria (such as neighborhoods in our case) may function well.^44^

Our study adds to a growing body of literature on neighborhood determinants of overweight and obesity in adults. In agreement with existing evidence, we showed that there are neighborhood-level factors that are associated with overweight and obesity, and these could be targeted in preventive strategies outside health care settings. The age of the residents appears to be highly relevant moderator as stronger and more consistent associations between environment and unhealthy weight were observed in older adults. Assuming that future research can confirm the causal character of such associations, the large effect sizes observed demonstrate the great potential of the neighborhood environment for addressing the problem of obesity and overweight. At the same time, individual demographic and socioeconomic characteristics remain important risk factors for obesity, and the population-level strategies could only be considered to complement the individual-level strategies to contain the obesity epidemic.

## Electronic supplementary material

Online appendix Table 1 Number of respondents per neighbourhood (DOCX 17 kb)

Online appendix Table 2 Association between neighborhood characteristics and overweight and obesity, stratified by education levelÂ£ (DOCX 18 kb)
