# Supplementary material for: Neighborhood Environment Is Associated with Overweight and Obesity, Particularly in Older Residents: Results from Cross-Sectional Study in Dutch Municipality
Source: J Urban Health. 2015 Oct 9;92(6):1038–51. doi: 10.1007/s11524-015-9991-y (PMC4675740; doi:10.1007/s11524-015-9991-y)
Supplement: Supplementary file 1 — Number of respondents per neighbourhood (DOCX 17 kb) [file 11524_2015_9991_MOESM1_ESM.docx]

Online appendix

Tabel 1. Number of respondents per neighbourhood

| **Neighbourhood name** | **Number of respondents** |
| --- | --- |
| City | 125 |
| Jekerkwartier | 86 |
| Kommelkwartier | 147 |
| Statenkwartier | 193 |
| Boschstraatkwartier | 140 |
| St. Maartenspoort | 155 |
| Wyck | 431 |
| Villapark | 387 |
| Jekerdal | 153 |
| Biesland | 238 |
| Campagne | 189 |
| Wolder | 232 |
| St. Pieter | 49 |
| Brusselsepoort | 405 |
| Mariaberg | 258 |
| Belfort | 392 |
| Pottenberg | 210 |
| Malpertuis | 205 |
| Caberg | 219 |
| Oud Caberg | 248 |
| Malberg | 284 |
| Dousberg-Hazendans | 221 |
| Daalhof | 443 |
| Boschpoort | 206 |
| Lanakerveld | 5 |
| Boscscherveld | 10 |
| Frontenkwartier | 24 |
| Wyckerpoort | 263 |
| Heugemerveld | 254 |
| Wittevrouwenveld | 252 |
| Nazareth | 218 |
| Limmel | 142 |
| Scharn | 483 |
| Amby | 450 |
| Borgharen | 214 |
| Itteren | 114 |
| Randwyck | 290 |
| Heugem | 355 |
| Heer | 416 |
| De Heeg | 358 |
| Vroendaal | 101 |
| Missing | 221 |
| **Total** | **9771** |
