# Supplementary material for: Neighborhood Environment Is Associated with Overweight and Obesity, Particularly in Older Residents: Results from Cross-Sectional Study in Dutch Municipality
Source: J Urban Health. 2015 Oct 9;92(6):1038–51. doi: 10.1007/s11524-015-9991-y (PMC4675740; doi:10.1007/s11524-015-9991-y)
Supplement: Supplementary file 2 — Association between neighborhood characteristics and overweight and obesity, stratified by education levelÂ£ (DOCX 18 kb) [file 11524_2015_9991_MOESM2_ESM.docx]

**Neighborhood characteristics and odds of overweight and obesity**

**Table 2. Association between neighborhood characteristics and overweight and obesity, stratified by education level£**

| Characteristic of neighborhood environment* | Overweight vs normal weight | Obese vs normal weight | Overweight vs normal weight | Obese vs normal weight | Overweight vs normal weight | Obese vs normal weight |
| --- | --- | --- | --- | --- | --- | --- |
|  | Odds ratio [95% CI]** | | | | | |
| Education | Low education **ⱡ** | | Secondary education **ⱡ ⱡ** | | Higher education **ⱡ ⱡ ⱡ** | |
| Reachability of facilities for daily use | 0.93 [0.86;1.00] | **0.89 [0.82;0.98]** | 0.93 [0.85;1.02] | 0.93 [0.82;1.05] | 0.95 [0.89;1.02] | 0.99 [0.87;1.13] |
| Traffic nuisance | 1.08 [0.99;1.19] | **0.89 [0.79;0.99]** | 1.05 [0.95;1.17] | 0.87 [0.75;1.01] | 1.08 [0.98;1.17] | 0.98 [0.84;1.15] |
| Neighborhood aesthetic (cleanliness) | 1.00 [0.69;1.45] | 0.73 [0.46;1.15] | **0.48 [0.31;0.73]** | **0.41 [0.23;0.75]** | 0.87 [0.65;1.15] | **0.53 [0.31;0.92]** |
| Nuisance by drunk people | 1.07 [0.98;1.17] | 1.02 [0.92;1.13] | 1.09 [0.99;1.19] | 1.10 [0.97;1.25] | **1.09 [1.03;1.16]** | 1.10 [0.99;1.23] |

*All aggregated indicators of neighborhood environment are scored 0 to 10, the higher the score, the better is the perception of the situation corresponding to the measured indicator

**Odds ratios per unit increase of the score, derived from multinomial logistic regression adjusted for individual gender and age group. Estimates with p-value<0.05 are highlighted **in bold**

**ⱡ** n= 3115 **ⱡ ⱡ** n= 2198 **ⱡ ⱡ ⱡ** n=3721

**£** stratified analyses are only performed when interaction term was significant (p<0.05)
